# Supplementary material for: Creating stable Floquet–Weyl semimetals by laser-driving of 3D Dirac materials
Source: Nat Commun. 2017 Jan 17;8:13940. doi: 10.1038/ncomms13940 (PMC5247574; doi:10.1038/ncomms13940)
Supplement: Peer Review File [file ncomms13940-s1.pdf]

Reviewers' comments:

Reviewer #1 (Remarks to the Author):

Novel topological phases and their realizations in materials, photonic and cold atom systems, etc. have been attracting increasing interest during the past few years. In particular, developing novel approaches for driving such systems across the topological phase transitions appear to be of the special interest. The authors of this manuscript propose an technique for transforming a Dirac semimetal phase into a Weyl semimetal or a topological insulator by means of ultrafast laser pulses of circularly polarized light. The suggestion is supported by means time-dependent DFT calculations performed on Na3Bi, an established Dirac semimetal material. I consider this result as highly important. In addition, the paper report valuable methodological developments in time-dependent DFT. I do not have any major critical remarks on this paper. However, I would like to see a stronger connection of the reported calculations with experiments that can be used for verifying the predictions. Once this issue is addressed, I will be able to recommend this manuscript for publication in Nature Communications.

Reviewer #2 (Remarks to the Author):

Dear Editor,

In their manuscript, they authors perform ab-initio calculations to numerically investigate the possibility to induce out-of-equilibrium analogs of a Weyl semi-metals and of (topological) band insulators from an equilibrium Dirac semi-metal, Na3Bi. Such a transition is found to be driven by a laser field whose periodic dependence in time can be tackled within Floquet theory which yields a quasi-energy band structure thus replacing the usual energy band structure of time-independent systems. This work falls within the interface of two very active fields: Floquet topological phases and topological Weyl semi-metals.

The main claim of the paper is that stable Floquet-Weyl points can be obtained and manipulated by the laser field parameters (such as the amplitude) despite crystalline symmetry breaking (in particular C3 rotation symmetry) which opens a gap in the equilibrium energy spectrum.

As recalled in the introduction, Weyl semi-metals (as well as topological insulators) have been observed experimentally. The proposition to obtain and manipulate such states of matter, (i.e. with a similar Floquet band structure) by an external and controllable field is appealing but has been already discussed several times in the literature (ref 46 for 3d and e.g [1] for 2d). Besides, it is not surprising that a circularly polarized electric field may drive a 3D Dirac semi-metal to a 3D Weyl semi-metal since time-reversal symmetry is broken and thus does not protect the 4-fold degeneracy. Also, the fact that a Weyl phase may exist while C3 symmetry is broken does not seem surprising neither, since first, C3 is not required to stabilize the Weyl phase, and second, it is expected that a Weyl phase necessarily exists between two insulating phases in 3D if time-reversal symmetry is broken [2]. The discussion about the role of the polarization to move the Weyl points away in "energy" or momenta (rather than horizontally and vertically which is ill-defined in p.8) is useful but constitutes, in my opinion, a minor result.

The "dancing" Floquet-Weyl points are maybe interesting but their discussion creates confusion: Are the trajectories of the Weyl points in Fig. 3B shown over one driving-period ? As the authors recall that the power of Floquet theory is to map a time-dependent problem onto a static problem (see Methods) how can the Floquet-Weyl points move in time? Besides, how does the filling of the bands follow this fast motion? The same question also arises when the authors claim, page 11, that "this light-induced phase transition might also be of technological relevance, because it facilitates light-controlled, ultra-fast switching between an insulating and a metallic phase". It seems to me that any statement about a metallic or an insulating state would require first to precise how the bands are filled, which is not obvious and certainly depends on the dissipation mechanisms [3]. Thus, one can emit doubts on the fact that proposing an out-of-equilibrium analog of Weyl semi-metals really helps in the quest of the observation of exotic phenomena such as "chiral magnetic effect" (page 12) whereas it is already challenging to observe in the equilibrium case. This is a legitimate perspective, but the manuscript does not really contribute to progress into that direction.

Finally, this study, as others, was led in a high-frequency limit. It is sometimes pointed out that such a limit, despite its practical convenience, is debatable, in particular as it uses models (low energy continuous Dirac/Wey equations or tight-binding models) which by construction (or by approximation) ignore bands at energies comparable to the photon-pulsation. It would have been very useful if the authors, which manage ab-initio techniques, could discuss the fate of higher bands and then justify or refute predictions made within models at low energy. Indeed, it is really not clear which results of the paper are derived from the low energy model (Eq. 8) and which are obtained from TDDFT. One can also regret that very few details are given about the "new framework of Floquet TDDFT" method that they authors use.

To conclude, I am not really convinced by the novelty, the originality and the capacity of this manuscript to significantly influence the field and the search of novel Weyl-Floquet phases. I thus cannot recommend it for publication in Nature Communications.

References:
[1] Phys. Rev. B 88, 245422 (2013)
[2] 2007 New J. Phys. 9 356
[3] Phys. Rev. X 5, 041050 (2015)

Reviewer #3 (Remarks to the Author):

The authors analyze the possibility to create a Floquet-Weyl semimetal by applying electromagnetic pulses to a Dirac semimetal---in particular Na_3Bi. They argue that by a proper choice of the pulse polarization, the Dirac cones can be split and moved in a well define manner in the Brillouin zone during the action of the pulse, leading to a topological non-equilibrium state.
The study is carried out using time-dependent Density Functional Theory in the framework of Floquet theory. This allows them to calculate the dressed band structure from first principles and to compare it with the ones of a more simple model.

The manuscript is very timely as the subject corresponds to a very active field of research. I do not think the manuscript proposes a new idea, but certainly uses an interesting methodology.
However, there are, in my opinion, several points that the authors must clarify/address for the manuscript to be considered for publication in the Nature Comm. I list them now:

a) The actual treatment of the time dependent perturbation is a bit unclear. On the one hand, Fig 3

shows the steering of the Weyl points as a function of the delay between pulses of different polarization, so clearly the calculation should be done with a finite pulse, is it?. On the other hand, all the rest of the figures, as well as the description of the method, is based on the Floquet theory, which in principle requires the perturbation to be periodic (not a pulse). I understand that the Floquet bands might correspond to what happens at the middle of the pulse, but then should not one worry about occupations (excited states created by the pulse itself), relaxation effects and several other minor effects? I think these points requires some particular clarification as the authors make some emphasis on the ab-initio character of the calculation.

b) I do not feel that the phrase "we establish the concept of time-dependent bands..." and similar ones is correct. The idea of an effective (dressed) band is already present in the literature.

c) Being the KS equations a set of self-consistent non-linear equations, is it clear that the period of the KS Hamiltonian (at the center of the finite pulse) will be set only by the external perturbation?

d) The authors claim that it is important to use their time dependent DFT approach since there are effects (dipolar transitions) that cannot be captured by a simple Peierls substitution in a tight-binding model. Please explain and quantify.

e) Related to the first point, I think that the phrase "After transient effects have decayed..." requires a more clear statement about time scales and the validity of the assumption for an actual pulse duration.

f) Can the authors elaborate more about the effect of high order replicas and how other dancing Floquet-WPs might appear?

g) In the methods sections the authors mention that they observe convergence of the effective Floquet bands with only the first order term. Please quantify.

h) I believe there is a typo in the definitions of the h_i parameters after Eq. (9)

Reviewers' comments:

Reviewer #2 (Remarks to the Author):

In their response to my comments, the authors have provided clear and sound statements which convinced me in the validity and the usefulness of their work for the search of topological out of equilibrium phases in real materials.
I don't have any other strong criticism and thus consider positively their manuscript for publication in nature comm.

Reviewer #3 (Remarks to the Author):

In their response the authors have reply to all my comments as well as those raised by the other referees. While I certainly believe that this manuscript is correct and certainly a valuable contribution to the field, I am not fully convinced by the authors' arguments that this manuscript is novel enough and/or has the potential to impact the field as to warrant publication on the Nat Comm. Therefore, I do not recommend it for publication.

A few comments

--It never was my point to doubt that, for instance, the tr-ARPES spectrum could be described by Floquet Theory. My point was that the method, at least as it was presented on the first version of the manuscript, did not handle 'pulsed fields' but rather used Floquet theory under the assumption that the time scale of the pulse envelop is much larger than the period of the electromagnetic radiation. I understand now that the method could, in principle, handle a real pulse, thought it remains unclear to my if that would represent a significant computational cost.

--In their reply the author provide a comparison of the ARPES spectrum obtained using their TDDFT method and some other unspecified method that uses real finite size pulses. Such comparison is rather useless for me if the other method is not specified at all: is it also an ab-initio method or is it one base on a tight-binding model? what determines the amplitude of the signal (color scale)? is their method able to capture that? In what sense is the TDDFT method better?

--The authors claim that after a 'stationary state' is reached the electronic structure must oscillate in phase with the driven field. I agree, but what time scale determines the 'stationary state' when the pulse is turned on? In addition it is mentioned that the Floquet regime is reached after two cycles. What do the authors mean by that? How is it quantified?

– I think that the fact that the TDDFT method captures some effects beyond the tight-binding model (asymmetric band splitting, according to the authors) should be explained better and not simply stated. As I pointed out in my first report, the novelty of the method was, in my opinion, the main reason why I believed the manuscript could be suitable for Nat. Comm.

--I also find the reply to the other referee's report a bit unsatisfying. I do not think that the fact that this method is applied to a 'real material' is enough to warrant publication in Nat. Comm.. I certainly think it is valuable contribution but being applied to a system that it very well described by a tight-binding model in a regime where where no potential surprises are expected, finding precisely that is not too exciting.

Reviewer #1 (Remarks to the Author):

Novel topological phases and their realizations in materials, photonic and cold atom systems, etc. have been attracting increasing interest during the past few years. In particular, developing novel approaches for driving such systems across the topological phase transitions appear to be of the special interest. The authors of this manuscript propose an technique for transforming a Dirac semimetal phase into a Weyl semimetal or a topological insulator by means of ultrafast laser pulses of circularly polarized light. The suggestion is supported by means time- dependent DFT calculations performed on Na3Bi, an established Dirac semimetal material. I consider this result as highly important. In addition, the paper report valuable methodological developments in time-dependent DFT. I do not have any major critical remarks on this paper. However, I would like to see a stronger connection of the reported calculations with experiments that can be used for verifying the predictions. Once this issue is addressed, I will be able to recommend this manuscript for publication in Nature Communications.

We thank the reviewer for his/her favourable view of our work and his recommendation for publication upon expanding our discussion of experimental verification. We have added to our discussion:

> Our predictions can be directly checked in pump-probe angle-resolved photoemission experiments, which can measure the transient Floquet band structures\cite{wang_unique_2013,mahmood_selective_2016}. These experiments could additionally even provide evidence about topological features implied by topological insulator, Dirac semimetal and Weyl semimetal materials, e.g. Fermi arc surface states of Weyl semimetal or surface states in topological insulators. Furthermore, a recent work\cite{lolodrubetz_non-adiabatic_2016} proposes measurement of the Wigner distribution as a means to identify non-trivial Floquet phases.

We would like to point out that Floquet theory is not a tool of convenience in the description of pump-probe ARPES experiments, but that is necessary to correctly describe the time-averaging that occurs by probing a system with a finite pulse duration. Therefore, we present here, for the benefit of the reviewer, confidential, unpublished results that illustrates this point. With a completely different method we have simulated the processes of photoemission from a semiconductor (WSe2) surface under resonant pump conditions with realistically shaped pump and probe pulses. The result is a fully ab initio calculated photoelectron spectrum of the pumped system:

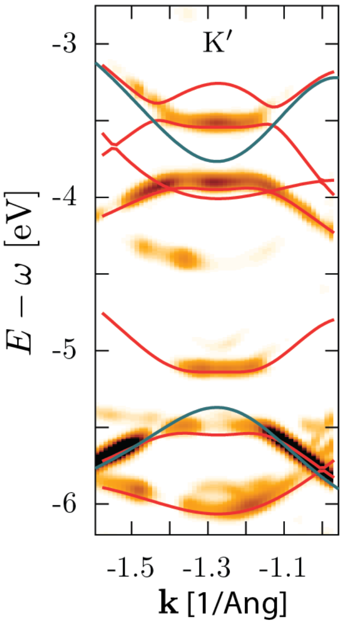

Figure 1: Ab initio ARPES spectrum of WSe2 at the K'-point pumped with energy resonant to the bandgap. Overlayed in green are the groundstate valence and conduction band, while in red is shown the ab initio Floquet bandstructure. The Floquet bands perfectly describe the hybridization effect (optical Stark effect) observed in the photoelectron spectrum.

The ARPES calculation makes no assumption whatsoever on periodicity, stationary states, etc., but the resulting spectrum perfectly agrees with the TDDFT-Floquet bandstructure, where no pulse shape is assumed. We stress that these are confidential results of a forthcoming publication[1], and that a similar calculation for NaBi is out of scope for this manuscript as well as unfeasible at the time for technical reasons.

[1] U. de Giovannini, H. Hübener, M. Sentef and A. Rubio (in preparation)

Reviewer #2 (Remarks to the Author):

Dear Editor,

In their manuscript, the authors perform ab-initio calculations to numerically investigate the possibility to induce out-of-equilibrium analogs of a Weyl semi-metals and of (topological) band insulators from an equilibrium Dirac semi-metal, Na3Bi. Such a transition is found to be driven by a laser field whose periodic dependence in time can be tackled within Floquet theory which yields a quasi-energy band structure thus replacing the usual energy band structure of time-independent systems. This work falls within the interface of two very active fields: Floquet topological phases and topological Weyl semi-metals.

We thank the reviewer for emphasizing one of the aims of this work by bridging two fields that are currently showing very fast development and huge research activity. This highlights the timeliness and potential impact of the current manuscript.

The main claim of the paper is that stable Floquet-Weyl points can be obtained and manipulated by the laser field parameters (such as the amplitude) despite crystalline symmetry breaking (in particular C3 rotation symmetry) which opens a gap in the equilibrium energy spectrum.

While being an important point of manuscript, the stability of Floquet-Weyl point is not our major claim, since it is a result that directly follows from the fundamental properties of Weyl-Fermions as pointed out by the reviewer below. Instead, the main claim of the paper is that we demonstrate that such non-equilibrium phases are indeed observable in real materials and with realistic pump field parameters.

As recalled in the introduction, Weyl semi-metals (as well as topological insulators) have been observed experimentally. The proposition to obtain and manipulate such states of matter, (i.e. with a similar Floquet band structure) by an external and controllable field is appealing but has been already discussed several times in the literature (ref 46 for 3d and e.g [1] for 2d).

While the general idea of light-induced topological Floquet-states in materials is not new, they have been mainly discussed for 2D systems (as pointed out by the reviewer). The few proposals for three dimensions, which is a necessary condition for the existence of Weyl fermions, are for

model systems rather than real materials, while here we demonstrate exactly this point, that they are observable in real materials as well.

```
Besides, it is not surprising that a circularly polarized electric
field may drive a 3D Dirac semi-metal to a 3D Weyl semi-metal since
time-reversal symmetry is broken and thus does not protect the 4-fold
degeneracy. Also, the fact that a Weyl phase may exist while C3
symmetry is broken does not seem surprising neither, since first, C3
is not required to stabilize the Weyl phase, and second, it is
expected that a Weyl phase necessarily exists between two insulating
phases in 3D if time-reversal symmetry is broken [2].
```

The reviewer makes the point that the proposed mechanism of non-equilibrium phase transitions are not surprising, which is certainly true when considering model materials which are designed to display the proposed effect. Indeed, the physics we discuss in the current manuscript follows from the Dirac equation formulated for electrons in a solid state system. However, it is far from obvious that the creation of a Floquet-Weyl semimetal is possible at all in a real material and so far none has been proposed or investigated.

```
The discussion about the role of the polarization to move the Weyl
points away in "energy" or momenta (rather than horizontally and
vertically which is ill-defined in p.8) is useful but constitutes, in
my opinion, a minor result.
```

The vertical movement of Floquet-Weyl points is indeed a new result which follows from the asymmetry in the bandstructure in kz-direction around the 3D Dirac point. This implies that in such real 3D-Dirac semimetals, as discussed here with NaBi, the non-equilibrium Floquet phases display a rich behaviour (metal, semi-metal, insulating) depending on the pump field. It highlights the necessity to move beyond model systems and investigate in detail the electronic structure and their excitation, of actual materials, as we have carried out here, for the first time in the context of Floquet-topological phases. We thus agree that by itself it is not a major result, but we think it is an important feature of 3D Dirac semimetals that has previously not been discussed and thus merits being included in the manuscript.

```
The "dancing" Floquet-Weyl points are maybe interesting but their
discussion creates confusion: Are the trajectories of the Weyl points
in Fig. 3B shown over one driving-period ? As the authors recall that
the power of Floquet theory is to map a time-dependent problem onto a
static problem (see Methods) how can the Floquet-Weyl points move in
time?
```

We thank the reviewer for pointing out the interest of our proposal to control the position of Floquet-Weyl points in the Brillouin zone, and we concede that the discussion in the text is a bit short. We have thus changed the relevant paragraph to explain the concept more clearly. The new paragraph now reads as:

> In a pump-probe experiment there are two different time-scales at play, the time of the period of oscillation and the time of the modulation of the amplitude due to the shape of the pump-pulse. The pump duration is typically order of magnitude longer than the oscillation of the field and hence the pulse shape has no effect on the formation of Floquet-Weyl points at any given time during the pumping. In our calculations we observe that the Floquet limit is reached after two cycles of the driving field, and thus this assumption holds even for relatively short pulses of 100 fs. However, the changing of the envelope amplitude over time will change the position of the Weyl points according to Fig. 2. This means that on the time-scale of the pump-envelope there is a movement of the Weyl points through the Brillouin zone. Furthermore, from the previous discussion it has become clear that combining different pump polarisation offers the possibility to control the position of the Floquet-WPs within the Brillouin zone. Thus, by modulating the amplitudes of two pump fields, i.e. by controlling the delay between two pump pulses, one can time-dependently steer the Floquet-WPs. Fig. 3 shows how a simple time delay between two Gaussian pump pulses in the x-z and y-z planes controls complex trajectories of the two Floquet-WPs through the Brillouin zone. With more sophisticated combinations of different pump envelopes one can design complex dynamics of Floquet-WPs on the time scale of the pulse duration. Thus Floquet-TDDFT also provides a way of analysing time-dependent band structures, that are different from the instantaneous eigenvalues of the TDDFT Hamiltonian, and that can be measured using time-resolved photoemission spectroscopy, which has an intrinsic time-averaging procedure given by the probe pulse shape.

We think that this clarifies the points raised by the reviewer.

```
Besides, how does the filling of the bands follow this fast motion?
The same question also arises when the authors claim, page 11, that
"this light-induced phase transition might also be of technological
relevance, because it facilitates light-controlled, ultra-fast
switching between an insulating and a metallic phase". It seems to me
that any statement about a metallic or an insulating state would
require first to precise how the bands are filled, which is not
obvious and certainly depends on the dissipation mechanisms [3].
```

The central mechanism discussed in this works is the lifting of the 4-fold degeneracy at the 3D Dirac point by symmetry breaking fields at off-resonance. Since the driving is done off resonance this mechanism does not require population transfer to excited states, but rather the effect is due to an effective gauge field due to the driving laser. But the reviewer is right to mention that the concepts of metallic and insulating phases out of equilibrium are not tied anymore to the original Fermi surface and that there might be competing relaxation mechanism at play. The focus here, however, is on the conditions required for the creation of the Floquet-Weyl semimetallic phase.

`Thus, one can emit doubts on the fact that proposing an out-of-equilibrium analog of Weyl semi-metals really helps in the quest of the observation of exotic phenomena such as "chiral magnetic effect" (page 12) whereas it is already challenging to observe in the equilibrium case. This is a legitimate perspective, but the manuscript does not really contribute to progress into that direction.`

In the discussion part of the manuscript we mention transport phenomena as a possible means of confirming the topological nature of the Floquet-Weyl phase beyond a possibly challenging measurement of the the non-equilibrium bandstructure. The fact that the whole field of non-equilibrium phase engineering and Floquet-topology is still in its infancy, does not, in our opinion, constitutes an argument against the relevance of our work, but to the contrary underlines its timeliness.

`Finally, this study, as others, was led in a high-frequency limit. It is sometimes pointed out that such a limit, despite its practical convenience, is debatable, in particular as it uses models (low energy continuous Dirac/Wey equations or tight-binding models) which by construction (or by approximation) ignore bands at energies comparable to the photon-pulsation.`

`It would have been very useful if the authors, which manage ab-initio techniques, could discuss the fate of higher bands and then justify or refute predictions made within models at low energy. Indeed, it is really not clear which results of the paper are derived from the low energy model (Eq. 8) and which are obtained from TDDFT.`

To remove any confusion and to dispel any the doubts of the reviewer on the generality of the high-frequency expansion we have substantially changed our manuscript such that we no longer resort to this expansion and all our results are obtained by using the full multi-photon

TDDFT-Floquet Hamiltonian. We feel that this also strengthens the point we make on the generality of the discussed phenomena as well as underlining the fact that with this work we present the first investigation of Floquet-topological phases by a fully ab initio approach and for a real material. In the text we only use the high-frequency expansion for the illustrative minimal model system that we present to explain the observed phenomenon. We have tried to make this as clear as possible in the text, for example by adding the sentence:

"To illustrate the basic idea behind the concept of Floquet-Weyl semimetal we briefly discuss the minimal model in which Floquet-WPs arise from a 3D Dirac point and then move to a fully ab-initio description of the real materials Na3Bi"

One can also regret that very few details are given about the "new framework of Floquet TDDFT" method that they authors use.

We feel that the outline of our method sufficiently explains the extension of the usual real-time TDDFT to the Floquet realm. We agree that we have not given much detail on TDDFT itself, but this would be beyond the scope of this work, especially as there is abundant literature on this topic.

To conclude, I am not really convinced by the novelty, the originality and the capacity of this manuscript to significantly influence the field and the search of novel Weyl-Floquet phases. I thus cannot recommend it for publication in Nature Communications.

References:
[1] Phys. Rev. B 88, 245422 (2013)
[2] 2007 New J. Phys. 9 356
[3] Phys. Rev. X 5, 041050 (2015)

Reviewer #3 (Remarks to the Author):

The authors analyze the possibility to create a Floquet-Weyl semimetal by applying electromagnetic pulses to a Dirac semimetal---in particular Na_3Bi. They argue that by a proper choice of the pulse polarization, the Dirac cones can be split and moved in a well define manner in the Brillouin zone during the action of the pulse, leading to a topological non-equilibrium state.
The study is carried out using time-dependent Density Functional Theory in the framework of Floquet theory. This allows them to calculate the dressed band structure from first principles and to compare it with the ones of a more simple model.

The manuscript is very timely as the subject corresponds to a very active field of research. I do not think the manuscript proposes a new idea, but certainly uses an interesting methodology.
However, there are, in my opinion, several points that the authors must clarify/address for the manuscript to be considered for publication in the Nature Comm. I list them now:

We thank the reviewer for his/her positive assessment of our work and for the detailed list of points he/she would like to have addressed in order to recommend publication in Nature Communications. Below we discuss these points in detail, along with the corresponding changes we made to the manuscript.

a) The actual treatment of the time dependent perturbation is a bit unclear. On the one hand, Fig 3 shows the steering of the Weyl points as a function of the delay between pulses of different polarization, so clearly the calculation should be done with a finite pulse, is it?. On the other hand, all the rest of the figures, as well as the description of the method, is based on the Floquet theory, which in principle requires the perturbation to be periodic (not a pulse). I understand that the Floquet bands might correspond to what happens at the middle of the pulse, but then should not one worry about occupations (excited states created by the pulse itself), relaxation effects and several other minor effects? I think these points requires some particular clarification as the authors make some emphasis on the ab-initio character of the calculation.

The reviewer correctly points out that Floquet theory requires the perturbation to be strictly periodic and hence has doubts about its applicability to real world pump-probe experiments, where the pump necessarily is a pulse with a finite envelope. It is, however, important to realize

that there are two different time scales involved here: the time scale of the envelope and the time of the period of the oscillation. The time period of a 1.5 eV (800nm) oscillation is 2.75 fs while a typical short pulse is of the order of 100fs. Thus there is a difference of about two orders of magnitude between theses two scales and hence one can make the assumption that the variation in amplitude due to the envelope has no bearing on the periodicity condition for Floquet theory. Indeed, the existence of the two different time scales of a pump-probe experiment is the whole point behind the idea of controlling the movement of Weyl points, because this movement occurs on the time scale of the envelopes.
To clarify this point we have changed the paragraph in the manuscript to:

> In a pump-probe experiment there are two different time-scales at play, the time of the period of oscillation and the time of the modulation of the amplitude due to the shape of the pump-pulse. The pump duration is typically order of magnitude longer than the oscillation of the field and hence the pulse shape has no effect on the formation of Floquet-Weyl points at any given time during the pumping. In our calculations we observe that the Floquet limit is reached after two cycles of the driving field, and thus this assumption holds even for relatively short pulses of 100 fs. However, the changing of the envelope amplitude over time will change the position of the Weyl points according to Fig. 2. This means that on the time-scale of the pump-envelope there is a movement of the Weyl points through the Brillouin zone. Furthermore, from the previous discussion it has become clear that combining different pump polarisation offers the possibility to control the position of the Floquet-WPs within the Brillouin zone. Thus, by modulating the amplitudes of two pump fields, i.e. by controlling the delay between two pump pulses, one can time-dependently steer the Floquet-WPs. Fig. 3 shows how a simple time delay between two Gaussian pump pulses in the x-z and y-z planes controls complex trajectories of the two Floquet-WPs through the Brillouin zone. With more sophisticated combinations of different pump envelopes one can design complex dynamics of Floquet-WPs on the time scale of the pulse duration. Thus Floquet-TDDFT also provides a way of analysing time-dependent band structures, that are different from the instantaneous eigenvalues of the TDDFT Hamiltonian, and that can be measured using time-resolved photoemission spectroscopy, which has an intrinsic time-averaging procedure given by the probe pulse shape.

We would like to stress the last point, that Floquet theory is not a tool of convenience in the description of pump-probe ARPES experiments, but that is necessary to correctly describe the time-averaging that occurs by probing a system with a finite pulse duration. Therefore, we present here, for the benefit of the reviewer, confidential, unpublished results that illustrates this point. With a completely different method we have simulated the processes of photoemission

from a semiconductor (WSe2) surface under resonant pump conditions with realistically shaped pump and probe pulses. The result is a fully ab initio calculated photoelectron spectrum of the pumped system:

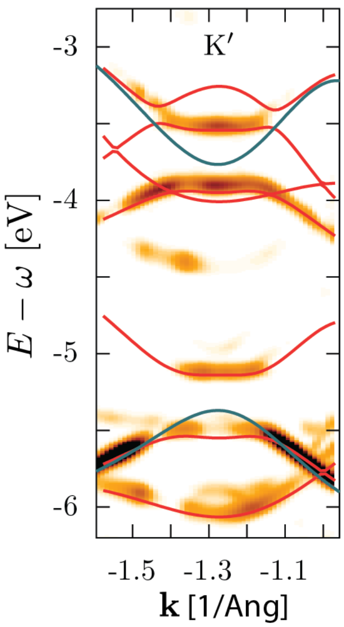

Figure 1: Ab initio ARPES spectrum of WSe2 at the K'-point pumped with energy resonant to the bandgap. Overlayed in green are the groundstate valence and conduction band, while in red is shown the ab initio Floquet bandstructure. The Floquet bands perfectly describe the hybridization effect (optical Stark effect) observed in the photoelectron spectrum.

The ARPES calculation makes no assumption whatsoever on periodicity, stationary states, etc., but the resulting spectrum perfectly agrees with the TDDFT-Floquet bandstructure, where no pulse shape is assumed.

We hope that this new results convinces the reviewer of the validity as well as the necessity of Floquet theory for the description of pump-probe experiments. But we stress that these are confidential results of a forthcoming publication[1], and that a similar calculation for NaBi is out of scope for this manuscript as well as unfeasible at the time for technical reasons.

b) I do not feel that the phrase "we establish the concept of time-dependent bands..." and similar ones is correct. The idea of an effective (dressed) band is already present in the literature.

We have removed such statements.

c) Being the KS equations a set of self-consistent non-linear equations, is it clear that the period of the KS Hamiltonian (at the center of the finite pulse) will be set only by the external perturbation?

When the time evolution has reached a stationary state the electronic structure has to oscillate in phase with the external driving field. This does not exclude the possibility of faster oscillations overlaying the driving period, but this is accounted for by the multiples of photon frequency in the Floquet expansion. Whether the Floquet limit can be reached with a finite pulse is a very valid question, but we find in our calculations that after two cycles this limit is reached, so that an experimental finite pulse with a FWHM of, say, more than ten cycles should certainly achieve this.

`d) The authors claim that it is important to use their time dependent DFT approach since there are effects (dipolar transitions) that cannot be captured by a simple Peierls substitution in a tight-binding model. Please explain and quantify.`

We do not mention these effects to justify the use of TDDFT, but rather to highlight some important differences with the commonly used model systems. The most important reason for using an ab initio method is that it provides a quantitatively reliable prediction of the time-evolution of the system without any fitting parameters. Tight-binding hamiltonians are usually developed to give an accurate ground state spectrum without constructing the wave functions. This has the added complication that the inclusion of the field in minimal coupling does not account for dipole transitions, or modifications of the assumed tight-binding wave functions. The TDDFT approach captures both of these effects.

`e) Related to the first point, I think that the phrase "After transient effects have decayed..." requires a more clear statement about time scales and the validity of the assumption for an actual pulse duration.`

The sentence in question is mentioned in the context of the toy model, where a quantitatve statement is not warranted. But we have included the sentence:

“In our calculations we observe that the Floquet limit is reached after two cycles of the driving field, and thus this assumption holds even for relatively short pulses of 100 fs.”

in the paragraph where we discuss the steering of the Floquet-Weyl points.

`f) Can the authors elaborate more about the effect of high order replicas and how other dancing Floquet-WPs might appear?`

While in this paper we only discuss the change of the ground state electronic bands, Floquet theory predicts the occurrence of sidebands, created by multi-photon processes. These sidebands

can also cross each other leading to secondary Floquet-Weyl points. If the primary Weyl points are moving around each other, so will these secondary ones in a synchronized movement. That said, Floquet sidebands have not yet been observed experimentally, so that the formation of secondary Weyl points, let alone their dancing, is not clear to be an actually observable phenomenon and therefore we chose to not discuss it further in the current work.

`g) In the methods sections the authors mention that they observe convergence of the effective Floquet bands with only the first order term. Please quantify.`

Since we are not using the perturbative approach in this manuscript we removed this statement. Instead, the only convergence parameter is the photon-dimension of the Floquet-Hamiltonian, and we have added the sentence

"In this work we found that the contributions of two-photon terms and beyond had a negligible effect on the bands considered here."

`h) I believe there is a typo in the definitions of the h_i parameters after Eq. (9)`

The equation in question has been removed.

[1] U. de Giovannini, H. Hübener, M. Sentef and A. Rubio (in preparation)

Reviewer #3 (Remarks to the Author):

In their response the authors have reply to all my comments as well as those raised by the other referees. While I certainly believe that this manuscript is correct and certainly a valuable contribution to the field, I am not fully convinced by the authors' arguments that this manuscript is novel enough and/or has the potential to impact the field as to warrant publication on the Nat Comm. Therefore, I do not recommend it for publication.

We thank the referee for finding our work correct and of value to the field and regret that he/she is not yet fully convinced by our arguments. However, we would like to point out that in his/her original report the referee conceded that: "... there are, in my opinion, several points that the authors must clarify/address for the manuscript to be considered for publication in the Nature Comm. " We believe that with our first revision and response to the referee to have addressed these points and further ones raised by the other referees. Nevertheless, we here gladly discuss the further points raised by the referee and make the corresponding changes in the manuscript.

A few comments

--It never was my point to doubt that, for instance, the tr-ARPES spectrum could be described by Floquet Theory. My point was that the method, at least as it was presented on the first version of the manuscript, did not handle 'pulsed fields' but rather used Floquet theory under the assumption that the time scale of the pulse envelop is much larger than the period of the electromagnetic radiation. I understand now that the method could, in principle, handle a real pulse, thought it remains unclear to my if that would represent a significant computational cost.

The referee is correct in assuming that our method can handle arbitrary general pulse envelopes. The only factor determining the computational cost attached to using them is the amount of time required until the system reaches the steady state. Besides, we have found that reaching a perfectly steady state is not even necessary for Floquet theory to be applicable and that small modulations of the driving amplitude do not substantially affect the Floquet bandstructure (c.f our preprint https://arxiv.org/abs/1609.03218).

--In their reply the author provide a comparison of the ARPES spectrum obtained using their TDDFT method and some other unspecified method that uses real finite size pulses. Such comparison is rather useless for me if the other method is not specified at all: is it also an ab-initio method or is it one base on a tight-binding model?

what determines the amplitude of the signal (color scale)? is their method able to capture that? In what sense is the TDDFT method better?

Both methods we discussed in the answer to the referee report are based on TDDFT. The technical details of the novel computational method we use to compute the ARPES spectrum is discussed in detail here: https://arxiv.org/abs/1609.03092 (submitted after the original submission of the present Nature Communications manuscript).
We discuss the relation between these two methods in another preprint here: https://arxiv.org/abs/1609.03218 where we show their remarkable agreeement and find that Floquet theory is applicable even when the periodicity condition is not strictly fullfilled. This is a very strong support for the existence of Floquet states in a TDDFT propagation. Floquet theory, as it is used in the present work, is just one way of analysing the dynamics and we mentioned the ARPES method as an alternative of accessing such states. Another option that is also within the capabilities of TDDFT, is probing the dynamics in an all optical pump-probe set-up, where signatures of the Floquet states manifest in the optical response. The theoretical description of such an experiment is ongoing work. Whether computed by direct Floquet analysis, or indirectly observed via simulation of a measurement process, the underlying photo-dressed Floquet states naturally emerge from our method of explicit time-propagation.

--The authors claim that after a ‘stationary state’ is reached the electronic structure must oscillate in phase with the driven field. I agree, but what time scale determines the ‘stationary state’ when the pulse is turned on? In addition it is mentioned that the Floquet regime is reached after two cycles. What do the authors mean by that? How is it quantified?

The regime where Floquet theory is applicable can be quantified by monitoring how the Floquet bandstructure changes during the propagation. When the system is in the steady state the Floquet bandstructure becomes static. The method section of the manuscript already describes this procedure by:

“Furthermore, the Floquet condition of periodicity does not have to be fulfilled a priori but one can perform the Floquet analysis over any time interval corresponding to the cycle of periodicity and determine the Floquet limit as a convergence of the Floquet bands with propagation time.”

- I think that the fact that the TDDFT method captures some effects beyond the tight-binding model (asymmetric band splitting, according to the authors) should be explained better and not simply stated. As I pointed out in my first report, the novelty of the method was, in my opinion, the main reason why I believed the manuscript could be suitable for Nat. Comm.

We made the following changes in the manuscript to clarify this point:

Line 58ff, before: “Importantly, our strategy goes beyond the use of model tight-binding Hamiltonians and light coupling via Peierls substitution, since the TDDFT scheme automatically deals with the electronic properties of the material and includes both Peierls phases for hopping terms and intra-atomic dipole transitions on equal footing.”

After: “Importantly, our strategy goes beyond the use of model tight-binding Hamiltonians and light coupling via Peierls substitution, since the TDDFT scheme automatically deals with the electronic properties and dynamical screening of the material and includes both Peierls phases for hopping terms and intra-atomic dipole (and higher multipole) transitions on equal footing. In fact our theoretical framework shows effects that are not captured by simple four band models. For example, the splitting of degenerate Dirac bands into bands supporting a Floquet-Weyl point under a pump field does not occur symmetrically in all cases as would be predicted by models and under linearly polarised pumping the dynamical electron-electron interaction can induce a symmetry breaking field that destroys the Dirac point and opens a gap.”

Line 68ff, before: “To illustrate the basic idea behind the concept of Floquet-Weyl semimetal we briefly discuss the minimal model in which Floquet-WPs arise from a 3D Dirac point and then move to a fully ab initio description of the real material Na3Bi.”

After: “To illustrate the basic idea behind the concept of dynamically driven Floquet-Weyl semimetal we briefly discuss the minimal model in which Floquet-WPs arise from a 3D Dirac point and then move to a fully ab initio description of the real material Na3Bi, where the dynamical electronic interactions of all valence electrons in the full Brillouin zone are taken into account.”

Line 122ff, before: “By solving the full ab initio mutli-photon Floquet-Hamiltonian without resorting to a high-frequency expansion or model parameters, we show that this effect occurs in real 3D Dirac semimetals, where due to asymmetry of the bands it can also lead to a non-symmetric shift of the two WPs in energy away from the Fermi level, one upwards and the other one downwards, forming a topological Weyl metal.”

After: “By solving the full ab initio mutli-photon Floquet-Hamiltonian without resorting to a high-frequency expansion or model parameters, we show that this effect occurs in real 3D Dirac semimetals and has the same dependence on amplitude and frequency but with a different prefactor. However, due to asymmetry of the bands in the actual material Na3Bi it can also lead to a non-symmetric shift of the two WPs in energy away from the Fermi level, one upwards and the other one downwards, forming a topological Weyl metal.”

Line 149ff, before: “In particular, we observe that the effect of asymmetric band splitting in the Weyl metal case requires dipole transitions, as it is not captured within the simpler model with Peierls substitution.”

After: “For example, the observed asymmetric band splitting in the Weyl metal case would not follow from a simple tight-binding model but would require including dipole transitions that are a priori unknown for such a model and would therefore have to be retrofitted.”

Line 159ff, before: “The Floquet-TDDFT framework introduced here will equally well apply to these situations.”

After: “The Floquet-TDDFT framework introduced here will equally well apply to these situations and many more.”

The differences between a tight-binding approach and a first principles calculation is not a matter of degrees but of scales. Tight-binding always needs to be justified by fitting to a given dataset and it is unlikely that it can reliably provide quantitative results beyond what was used to set up the model (limited range of applicability). As such they are useful in the development of conceptual ideas, but they require subsequent validation. Ab initio methods on the other hand are based on basic principles of quantum mechanics and carry predictive power and thus can provide precisely such a validation. We have added a paragraph to the method section in the manuscript (line 325ff) to clarify this point and why a full ab-initio calculation is needed:

“The fully ab initio nature of Floquet-TDDFT means that it requires no fitting reference. Instead, it might indeed be used as a reference itself for simplified qualitative tight-binding models that reduce the observed effect to its essential ingredients. For example one might think of using such an approach to refine the illustrative model we use in Eq. (1). While this can be useful in some case one, however loses the quantitative accuracy that TDDFT provides and in this case leads to quantitatively wrong dynamics of the Weyl points.”

--I also find the reply to the other referee’s report a bit unsatisfying. I do not think that the fact that this method is applied to a ‘real material’ is enough to warrant publication in Nat. Comm.. I certainly think it is valuable contribution but being applied to a system that it very well described by a tight-binding model in a regime where where no potential surprises are expected, finding precisely that is not too exciting.

We are sorry that the referee finds our work lacking excitement and concede that it is a matter of opinion. Tight-binding based approaches typically aim at only reproducing the band edges in a band structure and especially for Dirac materials focus on the Dirac point. The fact that in our approach, where we consider all valence electrons in the full Brillouin zone, without any fitting parameter while accounting for their full dynamics and interactions during time-propagation, we obtain a similar result to what the referee expects from tight-binding, is not so much a weak point of our method but rather a vindication of the widely used tight-binding approach. Experimental evidence of Floquet-topological effects is still scarce and our work represents the

transition of the field from toy-model conceptual approaches to a more mature materials science oriented direction, where the full properties of a real material are taken into account. We show that those are indeed needed to properly describe the electronic dynamics of the driven 3D Dirac semimetal that is not captured by the available tight-binding models.

In particular, our method accounts properly for electronic screening effects and for effects of laser driving both for driving electrons within one band (Peierls substitution) as well as for inducing local, intra-atomic dipolar transitions, and naturally contains all the relevant matrix elements. We note that such matrix elements would have to be included correctly in a tight-binding approach, which is not an easy task. Therefore we stress the superiority of our approach for describing optical driving effects in real multiband materials, as highlighted by the aforementioned discrepancies between TDDFT and the tight-binding model. In particular, a natural extension of the present method will be including the effects of nuclear motion (phonons) on the Floquet-electronic structure and even beyond that it provides a platform for directly studying the dynamical electronic-structure when driving coherent phonon modes with mid-IR laser pulses. Our study thus paves the way for studying a multitude of new effects expected to appear for optical driving in real materials.
